# Predominant Neck Extensor Muscle Weakness: A Rare Manifestation of Idiopathic Polymyositis

**DOI:** 10.7759/cureus.8735

**Published:** 2020-06-21

**Authors:** Hassan Kesserwani

**Affiliations:** 1 Neurology, Flowers Medical Group, Dothan, USA

**Keywords:** head drop, cervical extensor muscles, idiopathic polymyositis

## Abstract

Dropped head syndrome (DHS) is a rare disease. It can be an isolated phenomenon or secondary to an underlying inflammatory, genetic, or sporadic neuromuscular disease. Idiopathic polymyositis as an underlying cause of DHS is rare and this association has been described very few times in the literature. We describe a case of biopsy-proven polymyositis presenting with predominant neck extensor muscle weakness. This case report goes further into analyzing the biomechanics of neck extension and putative reasons for predilection of the neck extensor muscles with advancing age in patients with DHS who have underlying idiopathic polymyositis.

## Introduction

Dropped head syndrome (DHS) is a rare disease. It is due to the weakness of the neck extensor muscles. It can be painful, cosmetically disfiguring, interfere with swallowing and impair forward gaze. DHS can be isolated or secondary to an underlying disease. When isolated, DHS can be due to a biomechanical cause or an isolated extensor myopathy. There are many secondary causes including inflammatory myopathies, genetic myopathies, metabolic myopathies, myasthenia gravis, amyotrophic lateral sclerosis, and a wide range of rare associations [1-4]. Cervical dystonia can mimic DHS, but the latter is due to abnormal muscle tone, and not weakness of the extensor neck muscles. The natural history of DHS is progression to an abnormal fixed posture. Biomechanical causes can be amenable to surgical correction [5]. When inflammatory, it can respond to treatment of the underlying disorder with steroids or immunomodulatory agents [6]. A unique focal myopathy, leading to weakness of neck extensor muscles, known as isolated extensor myopathy, has been described. This may have a favorable response to steroids; however, it may be either be inflammatory or non-inflammatory myopathy [7-10]. There are very few reports of DHS secondary to biopsy-proven idiopathic polymyositis in the literature [9]. Idiopathic polymyositis is an inflammatory myopathy. It is due to clonally expanded cytotoxic CD-positive T- cells attacking and invading myocytes coated with major histocompatibility complex (MHC) class 1 antigens, leading to muscle fiber necrosis. Idiopathic polymyositis predominantly leads to progressive symmetric proximal muscle weakness [11]. It can be associated with bulbar weakness and interstitial lung disease. Rarely, it can be paraneoplastic. We present a rare case of biopsy-proven polymyositis presenting with prominent head drop and mild proximal weakness. The muscle biopsy reveals an intense inflammatory reaction with lymphocytes invading myocytes. However, due to the patient's brittle diabetes and religious faith, Jehovah's witness, our patient refused steroids and/or intravenous immunoglobulin (IVIg). Other options such as methotrexate, azathioprine, and rituximab were not appropriate as she had a low-grade idiopathic pancytopenia. The dropped head developed into a permanent flexed posture of the neck. This case report highlights the rare association of head drop and idiopathic polymyositis, and we explore the biomechanics of head drop and its predisposition with advancing age. We further hypothesize that fiber type switching from slow twitch type 1 to fast twitch type 2 with advancing age and in inflammatory myopathies may contribute to this odd and unique phenotype.

## Case presentation

**Figure 1:**
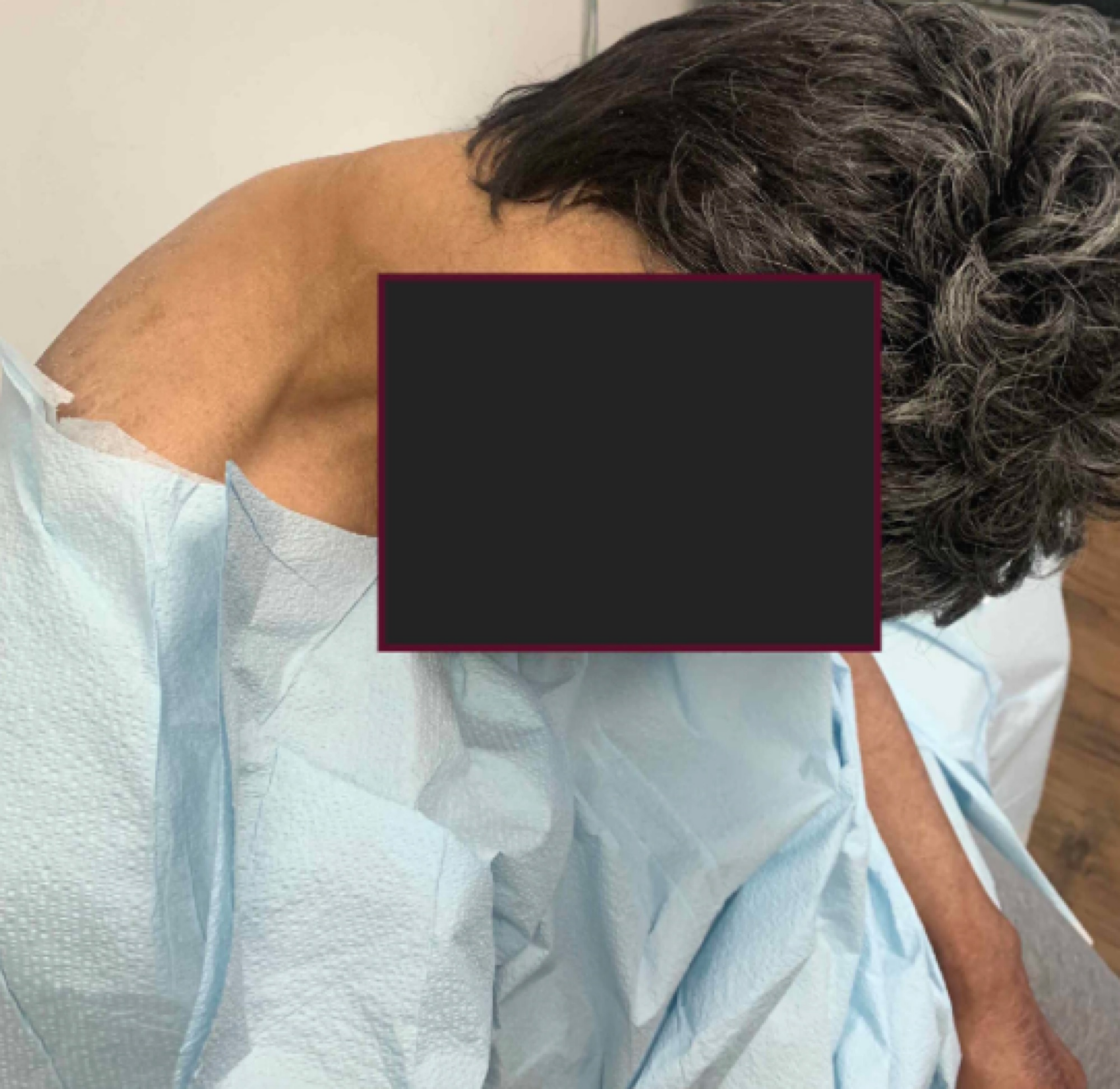
Head drop with atrophy of neck extensors

A 75-year-old woman presented with a six-month history of painless progressive head drop and mild weakness of the upper and lower extremities. She had developed difficulty maintaining frontal gaze while conversing. Her neck would tire and she adopted a head ptosis. She was able to challenge gravity with her arms and wear a shirt and she was able to reach above shoulder level. She had a long-standing history of poorly controlled diabetes and had an advanced diabetic neuropathy with anesthetic feet. She developed gait instability and ambulated with a walker for safety. She denied fatigability. She denied diplopia, dysarthria, or dysphagia. She had a complicated medical history with advanced diabetic triopathy: retinopathy, neuropathy, and nephropathy. She had coronary artery disease and low-grade idiopathic pancytopenia. Her medications included insulin, amlodipine, bumetanide, gabapentin, levothyroxine, metoprolol, and oxymetazoline. On examination, eye movements were full in all directions. There was no eyelid ptosis and no fatigability with repetitive blinking. She had no facial weakness. She had no hypophonia or dysarthria. She had an obvious head drop with atrophy and weakness of neck extensors (Figure 1). She was unable to raise her head and maintain frontal gaze. She could stand without assistance. She had a Romberg sign. She could take a few steps without a walker.

**Figure 2:**
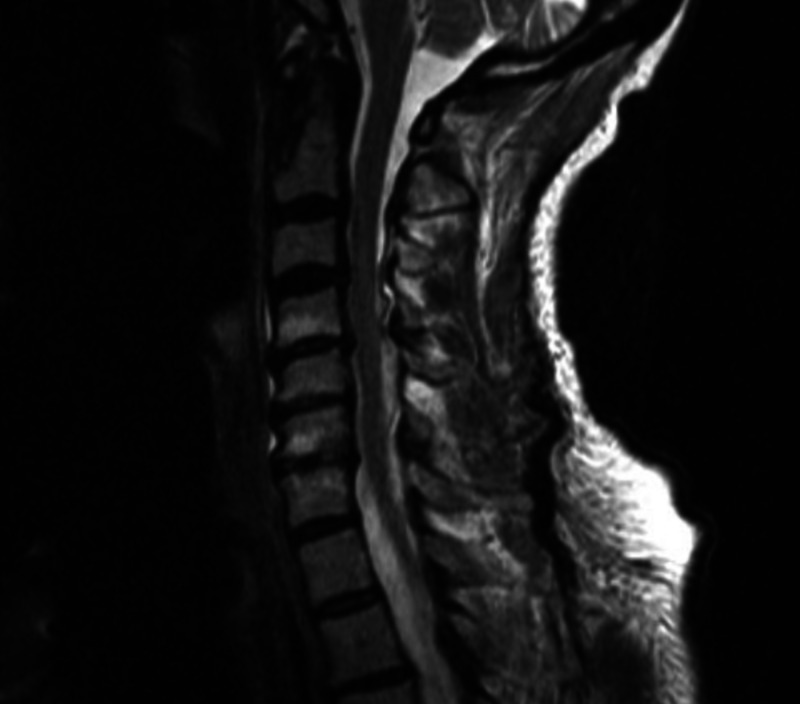
T2 weighted sagittal MRI of the cervical spine reveals moderately severe spondylosis but no kyphosis to explain head drop

Motor examination with Medical Research Council (MRC) grading scale revealed symmetric proximal weakness in the upper extremities; muscle strength was graded as deltoids 4/5 symmerically, biceps 5/5 symmetrically, brachioradialis 5/5 symmetrically, finger spreaders, thenars and hypothenars 5/5 symmetrically. In the lower extremities; iliopsoas 4/5 symmetrically, quadriceps 5/5 symmetrically, hamstrings 5/5 symmetrically, ankle dorsiflexors/plantar flexors 5/5 symmetrically. Sensory examination revealed anesthetic feet with loss of pain, simple touch, temperature, and loss of joint position sense of the toes. Deep tendon reflexes were absent in the legs, and diminished in the upper extremities. Serology included a creatine phosphokinase (CPK) of 2099 (> 269) and a negative myasthenia gravis panel that included acetylcholine receptor binding, blocking, and striational antibodies. An MRI of the cervical spine revealed moderate spondylosis but without any significant cord compression or kyphosis to explain the head drop (Figure 2).

**Figure 3:**
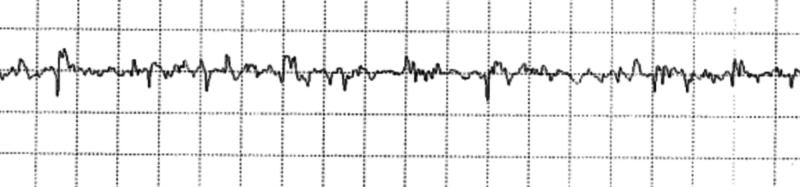
Electromyogram recording of left biceps muscle showing short duration, small amplitude, polyphasic motor units with full interference pattern; sensitivity 500 microvolt and base 10 milliseconds

A nerve conduction/electromyogram revealed myopathic units in the left deltoids, biceps, and neck extensors with early recruitment and full interference pattern (Figure 3).

**Figure 4:**
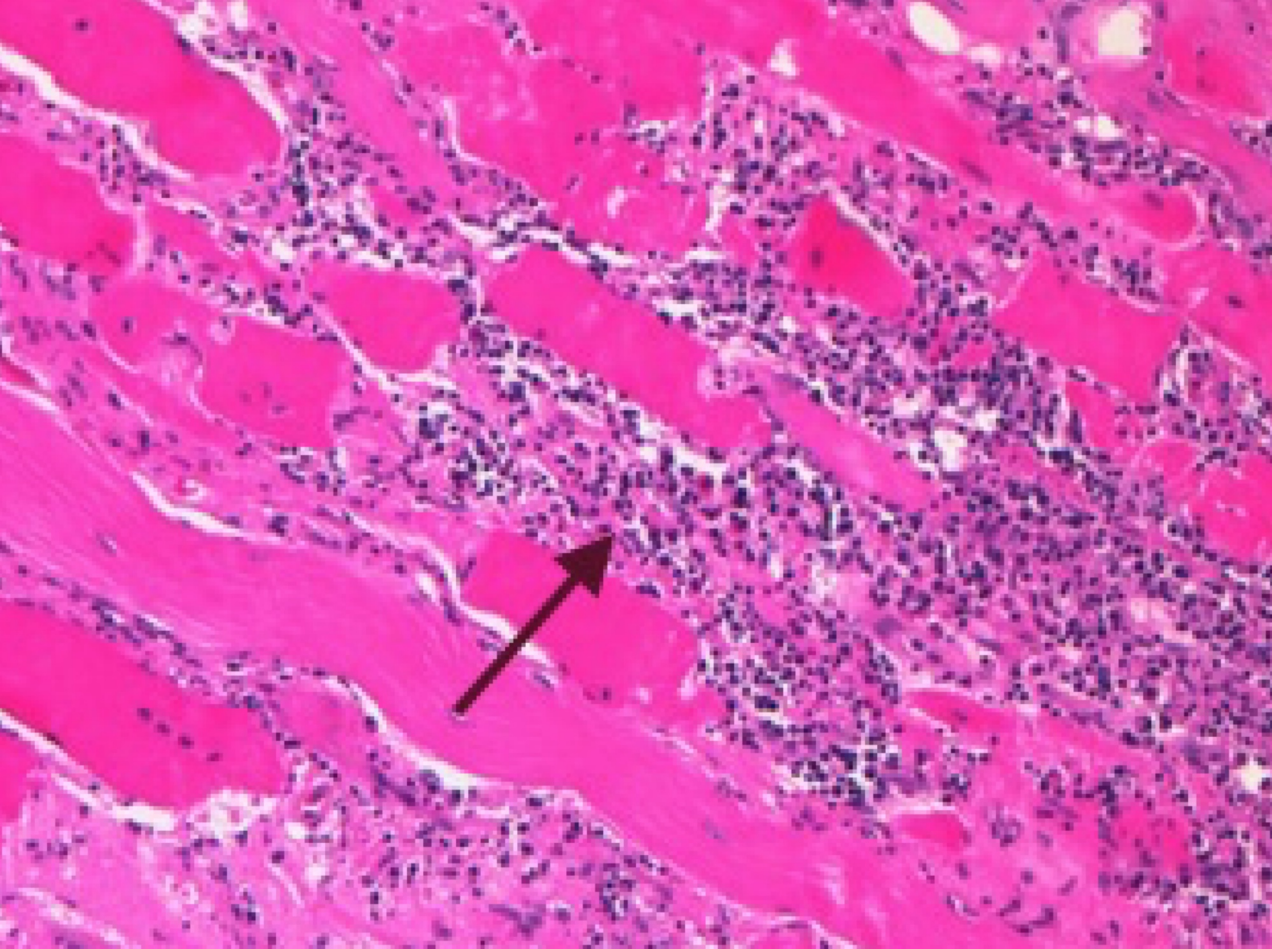
Hematoxylin and eosin stain of a longitudinal section demonstrating intense inflammatory invasion of a degenerated myocyte (arrow)

**Figure 5:**
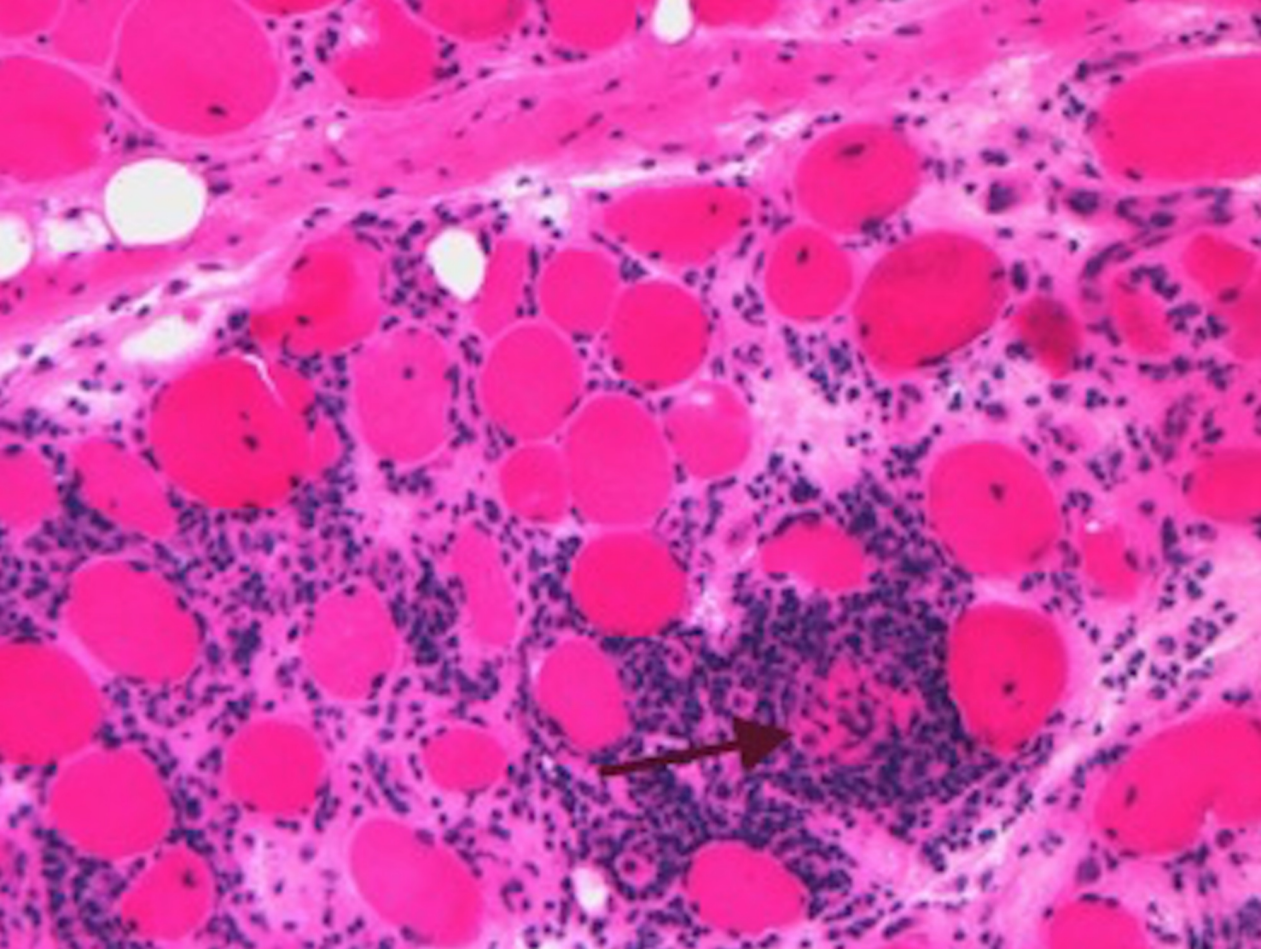
Hematoxylin and eosin stain demonstrating an axial section showing variation of muscle fiber size and central nuclei typical of a myopathy; an intense inflammatory invasion of a partially degenerated myocyte

A left deltoid muscle biopsy was obtained (Figures 4-5). This revealed variation of muscle fiber size, myocytes with central nuclei and an intense endomysial inflammatory infiltrate around degenerating myocytes, typical of idiopathic polymyositis. There was no evidence of rimmed vacuoles, granulomas, ragged red fibers or vasculitis.

A diagnosis of idiopathic polymyositis with mild proximal myopathy with predominant affliction of neck extensor muscle was made based upon clinical presentation, serology, and deltoid muscle biopsy findings. A course of steroids was denied by the patient on account of her advanced diabetic triopathy. IVIg was refused on grounds of her religious belief, Jehovah's witness. Methotrexate, azathioprine, and rituximab were deemed inappropriate on account of her idiopathic pancytopenia. Nevertheless, the patient remained optimistic and jovial despite her difficulties. She remained stable and independent in most of her activities of daily living. Stability was sustained four months after her initial presentation.

## Discussion

Why inflammatory or genetic myopathies may have a predilection for extensor neck muscles over flexor muscles is not entirely clear. A brief overview of cervical spine biomechanics and histology of the muscles may shed some light on this. The curvature of the cervical spine is called lordosis and the concavity faces posteriorly. The cervical spine supports the head which weighs approximately four kilograms. The movements of the cervical spine are a) flexion-extension, 50% of extension happens in the upper cervical spine, b) rotation, 50% of which happens in the C1-C2 level and, c) lateral flexion which happens mostly in the middle part of the cervical spine. The superficial extensor muscles are the trapezius which also elevates the shoulder, elevates the scapula and laterally flexes the neck and the levator scapula which mainly elevates the shoulder. The middle layer extensor muscles which include the splenius capitis and splenus cervicis which extend, laterally flex and ipsilaterally rotate the neck. The deep layer of muscles include the semispinalis capitis, rectus posterior major and minor and obliquus superior which also extends the neck, laterally flex, and ipsilaterally rotate the neck. These are anti-gravity muscles. They are mostly long, allowing for increased torque and an enhanced mechanical advantage. There are very few histological studies analyzing fiber typing of the neck extensor muscles. The trapezius muscle is a long muscle. The proximal section is at a mechanical disadvantage compared to the distal section as it lies closer to the fulcrum of flexion. This would impose different functional demands on different parts of the trapezius. Hence, one would expect the proximal muscle section to exert a more forceful torque than the distal sections, because of mechanical disadvantage, a shorter arm of the lever. One would also expect the proximal groups to be rich in fast twitch fatiguable type 2 muscle fibers and the distal groups to contain more slow twitch fatigue resistant type 1 muscle fibers. Indeed, this is borne out by histological studies [12]. 

There are two muscle fiber types. Type 1 muscle fibers are slow twitch and rich in mitochondria; they deploy aerobic metabolism. They are recruited in long endurance tasks such as anti-gravity muscles and muscles deployed for long-distance running. They are resistant to fatigue. Type 2 muscle fibers are fast twitch, rich in myoglobin, and deploy anaerobic metabolism. They are deployed during activities such as sprinting [13]. Aging leads to loss of both fiber types, with preferential atrophy of type 2 muscle fibers, and fiber type transformation; from slow twitch to fast twitch muscle fibers, that is, from type 1 fatigue-resistant to type 2 fatiguable muscle fibers. The trend in chronic inflammatory muscle diseases is a switch from slow to fast twitch muscle fibers. Slow-twitch fibres are in general more fatigue resistant than fast-twitch fibres because of their higher oxidative capacity [14].

Myocytes are a target of immune-mediated damage. Removal of immune cells from the milieu could result in the preservation of skeletal muscle cells which are able to recognize injury-associated self-proteins via pattern recognition receptors. These receptors recognize damage-associated molecular patterns (DAMPs), which are either derived from foreign antigens or self-proteins, stress or danger-associated molecular patterns (SAMPs). Recognition of DAMPs by toll-like receptors (TLRs) initiates a signaling cascade, leading to the activation or inhibition of genes that control the inflammatory response [15]. 

Type I fibers are more susceptible to inactivity and denervation atrophy, while type 2 fibers are more susceptible to cancer, diabetes, heart disease, and aging. This difference in susceptibility can be explained by the activation and response to different signaling pathways. The peroxisome proliferator-activated receptor gamma coactivator 1-alpha (PGC1α) protects type I fibers from atrophy while the transforming growth factor beta (TGFβ) family and the nuclear factor kappaB (NF-κB) predominantly affect type 2 fibers [16]. 

We hypothesize that the unique fiber typing of the neck extensor muscles, as illustrated by the trapezius, causes transformation from type 1 fatigue resistant muscle fibers to type 2 fatiguable muscle fibers with age and inflammatory muscle disease, and may predispose these muscles to stress, damage, and immune attack. This is speculative and requires further study.

Our patient had classic idiopathic polymyositis with prominent head drop. Invariably, these patients have a symmetric proximal myopathy. In our case, the proximal myopathy was mild. She has chronic illnesses including diabetes, chronic renal failure, and coronary artery disease. She was also advanced in age and inactive. These are all predisposing factors for fiber type transformation from slow to fast twitch fibers. The fast twitch fibers are fatiguable and when diseased are prone to immune attack due release of DAMPs and inefficient clearing pathways such as NF-kB and TLRs [15,16]. This is a putative mechanism for their predilection to immune attack and warrants further study.

## Conclusions

Our case is one of the rare examples of biopsy-confirmed DHS secondary to idiopathic polymyositis. This is noteworthy as idiopathic polymyositis is a potentially treatable disease. This is an odd and unique phenotype worthy of further study. While these and other inflammatory myopathies can rarely present with head drop, they invariably also involve the proximal muscle groups. We go further in this article and analyze the biomechanics of neck extension and their predilection to weakness with advancing age and inflammatory muscle disease. We hypothesize that fiber type switching from fatigue-resistant slow twitch fibers to fatiguable fast twitch fibers in the neck extensors predisposes them to immune attack when the interstitial milieu is conducive to immune attack, as seen with idiopathic polymyositis. This hypothetical speculation needs to be borne out by further studies in the future.
